# All‐Hydrogel‐Based Organic Electrochemical Transistors for Implantable Physiological Signal Monitoring

**DOI:** 10.1002/advs.202517375

**Published:** 2025-11-14

**Authors:** Qicheng Liang, Yue Wang, Ruizhe Wang, Wanfang Zhang, Runcheng Hao, Yueheng Zhong, Jingling Zhang, Xiang Li, Weichu Chen, Chunyu Fan, Yuwen Zhu, Yu Sun, Hong Jiang, Hengda Sun, Gang Wang

**Affiliations:** ^1^ State Key Laboratory of Advanced Fiber Materials College of Materials Science and Engineering Donghua University Shanghai 201620 China; ^2^ Department of Anesthesiology Shanghai Ninth People's Hospital Shanghai Jiao Tong University School of Medicine Shanghai 200011 China; ^3^ Henan Academy of Sciences Zhengzhou 450046 China

**Keywords:** implantable bioelectronics, n‐type semiconductor hydrogel, organic electrochemical transistor, physiological signal monitoring

## Abstract

Organic electrochemical transistors (OECTs) are an advanced technology for interfacing with biology, capable of efficiently transducing ionic currents into amplified electronic signals at low operating voltages. However, their clinical potential is critically undermined by the mechanical mismatch between conventional device materials and soft tissues. This fundamental limitation is resolved by developing a versatile all‐hydrogel‐based OECT, which is made possible by a novel n‐type depletion‐mode semiconductor hydrogel. Fabricated via an ionic liquid‐mediated phase separation of poly(benzodifurandione)/polyacrylamide, the material uniquely combines efficient ion‐electron coupled transport with tissue‐like softness. This fully compliant architecture delivers a high transconductance of 43 mS, remains stable over 1500 bending cycles, and shows excellent biocompatibility. The devices enable real‐time monitoring of human electrocardiography/electrooculogram (ECG/EOG) signals on the skin and long‐term subcutaneous recording of nociceptive/ECG signals in rats. This study provides a robust materials and device strategy, establishing a versatile platform for multimodal physiological signal monitoring, pain assessment, cardiovascular health evaluation, and implantable precision bioelectronic therapy.

## Graphical abstract

All‐hydrogel based n‐type depletion‐mode OECTs is developed using a semiconductor hydrogel formed via ionic‐liquid‐mediated phase separation, combining efficient ion‐electron transport, tissue‐like softness, and long‐term biocompatibility. The device can monitor EOG and ECG on human skin and record real‐time ECG subcutaneously in rats, providing a versatile platform for multimodal physiological sensing and implantable bioelectronics.

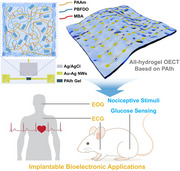

## 1 Introduction

Organic electrochemical transistors (OECTs), with their unique ion‐electron coupled conduction mechanisms, can transduce ionic signals into electronic signals via electrochemical processes.^[^ ^1^, ^2^, ^3^ ^]^ Their advantages—including low operating voltage, high transconductance, and environmental adaptability—enable highly sensitive identification and amplification of physiological signals, making them particularly promising for monitoring electrocardiography (ECG), electrooculogram (EOG), electromyography (EMG) and biomolecular signals.^[^ ^4^, ^5^, ^6^ ^]^

However, conventional OECTs are composed of conjugated polymer thin film, rigid electrodes, and hard substrates, and their modulus far exceeds that of biological tissues.^[^ ^7^, ^8^ ^]^ This mechanical mismatch at the tissue‐device interface can lead to inflammatory responses, signal attenuation, or device failure.^[^ ^9^, ^10^, ^11^, ^12^ ^]^ To address this, hydrogel materials have recently been integrated into OECTs due to their tissue‐mimetic mechanical properties and excellent ionic conductivity, thereby improving device‐tissue compatibility.^[^ ^13^, ^14^, ^15^, ^16^ ^]^ For instance, Wang et al. employed a solvent‐exchange method to convert the non‐water‐soluble semiconductor p(g2T‐T) into a hydrogel,^[^ ^17^ ^]^ achieving a modulus of 100 kPa and a carrier mobility of 1.4 cm^2^ V^−1^ s^−1^. Lei et al. synthesized an n‐type enhancement‐mode semiconductor and fabricated the hydrogel P(PyV) via ionic cross‐linking, enabling high‐gain logic circuits and electroencephalogram signal amplification,^[^ ^18^ ^]^ and Huang et al. focused on gelation the electrolyte to further improve device performance.^[^ ^19^ ^]^ Despite these advances, current hydrogel semiconductor fabrication methods face significant limitations. The solvent‐exchange approach applies only to semiconducting polymers with hydrophilic side chains, making it difficult to extend to a broader range of materials, and n‐type depletion‐mode semiconductor hydrogels remain largely unexplored. At the device level, research has predominantly targeted “partial gelation” with substrates and electrodes still composed of high‐modulus materials.^[^ ^12^, ^20^, ^21^, ^22^ ^]^ Moreover, the absence of reliable patterning leads to inconsistent contact areas between the semiconductor and electrodes and irregular interfacial boundaries, resulting in poor electrical uniformity and nonuniform strain distribution, which ultimately compromise device reliability under dynamic deformation and long‐term implantation.

To address these pressing challenges, we introduce an ionic liquid (IL)‐mediated phase separation strategy to create a novel n‐type depletion‐mode semiconductor hydrogel. Specifically, we engineer interconnected conductive networks of the poly(benzodifurandione) (PBFDO) within a soft polyacrylamide (PAAm) matrix, achieving a photo‐patternable n‐type depletion‐mode semiconductor hydrogel with excellent intrinsic conductivity, mechanical compliance closely matched to biological tissues, and favorable biocompatibility. Building upon this material innovation, we construct an all‐hydrogel based OECT by integrating the semiconductor hydrogel with nanowire electrodes and a poly(acrylic acid) (PAAc) hydrogel substrate. This design overcomes the multi‐interface modulus mismatch inherent to conventional OECTs, substantially enhancing mechanical compliance and biocompatibility, while also enabling reliable patterning and logic circuit fabrication. We demonstrate that this all‐hydrogel platform exhibits excellent biocompatibility, and enables long‐term, high‐fidelity physiological signal monitoring both on the skin surface and within deep tissue, thereby providing a transformative solution for advanced implantable bioelectronics.

## 2 Results

### 2.1 Preparation and Characterization of PAIh Semiconductor Hydrogels

**Figure 1:**
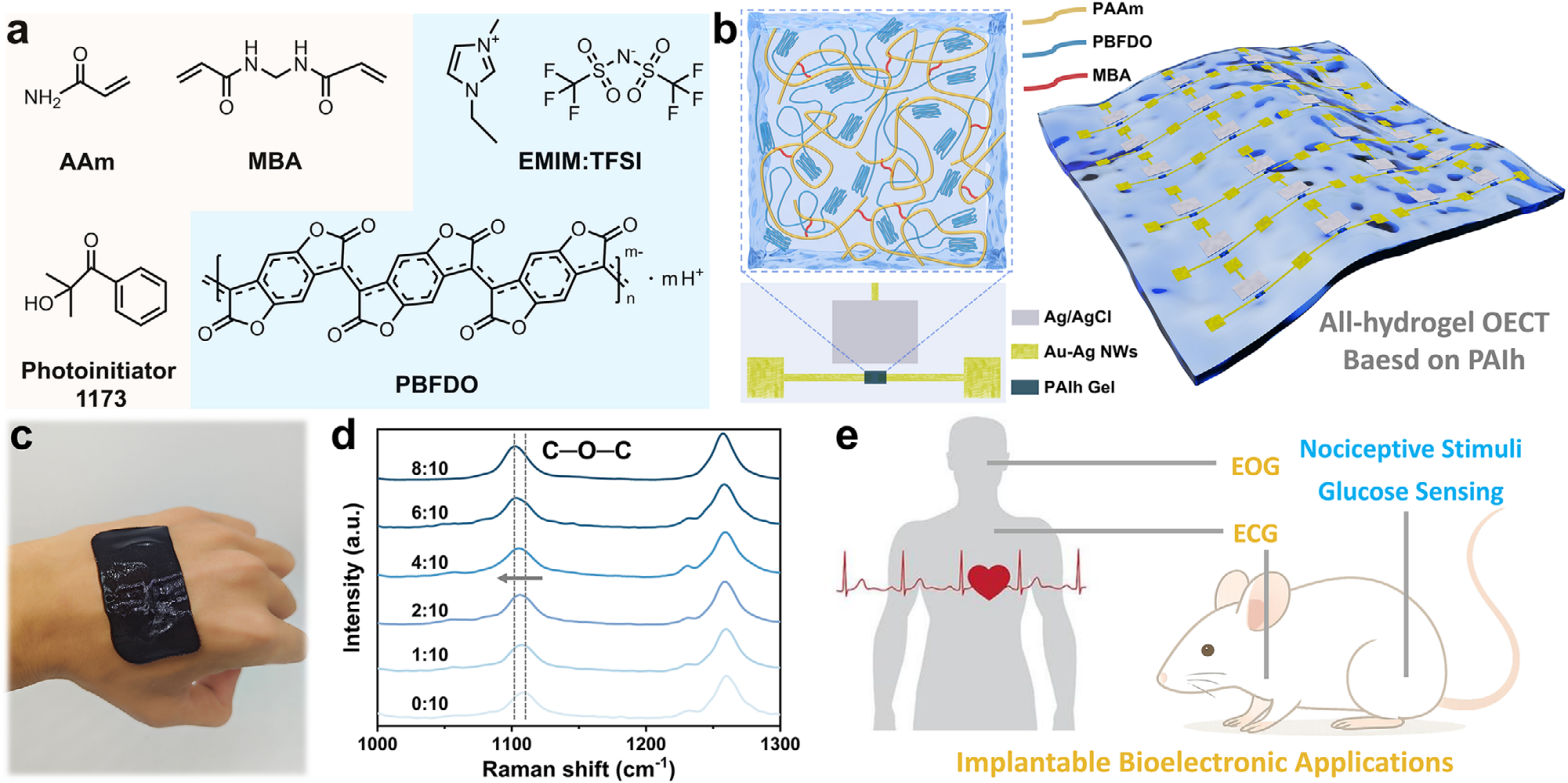
a) Composition of the precursor solution for preparing PAIh semiconductor hydrogels. b) Schematic illustration of the PAIh semiconductor hydrogel structure and the hydrogel‐based OECTs. c) Photograph of the PAIh semiconductor hydrogel adhered to the dorsal side of a human hand. d) Raman spectra of PAIh hydrogels with different IL‐to‐PBFDO mass ratios. e) Application of PAIh‐based hydrogel OECTs in implantable bio‐signal monitoring.

PBFDO is an n‐type depletion‐mode semiconductor polymer with high carrier mobility, chemical stability, and facile synthesis, widely used as the functional layer in OECTs.^[^ ^23^, ^24^, ^25^ ^]^ However, PBFDO exhibits poor solubility in water and dissolves only in polar aprotic solvents such as dimethyl sulfoxide, making direct hydrogel fabrication challenging. Exploiting the high miscibility of DMSO with water, an effective approach involves first preparing an organic gel in DMSO, followed by solvent exchange with water to produce a PBFDO‐based hydrogel. To this end, a commercial PBFDO solution was mixed with the conventional hydrogel monomer acrylamide (AAm), the crosslinker N,N′‐methylenebisacrylamide (MBA), the photoinitiator 2‐hydroxy‐2‐methylpropiophenone (Photoinitiator 1173), and the ionic liquid [EMIM]^+^ [TFSI]^−^ (IL) as an additive (**Figure** **1**a). The mixture underwent 365 nm UV‐induced free radical polymerization and cross‐linking, forming a PBFDO‐based organic gel, which was then subjected to solvent exchange in DI water to obtain the PBFDO/PAAm/IL hydrogel (PAIh). Due to its UV‐induced gelation, PAIh can be patterned into defined geometries on a substrate, achieving a minimum feature size of 50 µm (Figure S1, Supporting Information). This capability facilitates the construction of the hydrogel OECTs on substrates through controllable patterning methods.

PAIh hydrogels with varying IL content were prepared for comparative analysis. Interactions among PAIh components were characterized by Raman spectroscopy. Figure S2 (Supporting Information) shows the Raman spectra of pristine PBFDO and PBFDO/PAAm hydrogels without IL. Peaks at 1510 and 1259 cm^−1^ correspond to the stretching vibrations of C═C in the conjugated backbone and the in‐plane bending of C─H, respectively, while peaks at 1110 and 1623 cm^−1^ are attributed to C─O─C and C═O stretching vibrations.^[^ ^26^ ^]^ Upon incorporation of PAAm, the Raman peaks of C─O─C and C═O of PBFDO exhibited a redshift (shifting from 1110 to 1108 cm^−1^ and from 1623 to 1619 cm^−1^, respectively), indicating hydrogen bond formation between PBFDO and PAAm chains, which reduces vibrational energy. To examine the effect of IL on the hydrogel system, Raman spectra of PAIh with different IL contents were recorded (Figure 1d). Increasing IL content caused a pronounced redshift of the C─O─C peak from 1108 to 1102 cm^−1^, resulting from electrostatic interactions between IL and PBFDO that weaken the vibrational frequency and intensity of covalent bonds. These suggest IL‐PBFDO interactions induce phase separation in PAIh, promoting the formation of interconnected conductive domains/pathways and consequently affecting the gel's electrical and mechanical properties.^[^ ^27^ ^]^ Atomic force microscopy (AFM) images revealed that the incorporation of IL led to increased surface roughness in the semiconductor hydrogel films, accompanied by phase formation (Figure S4, Supporting Information). Conductive AFM (C‐AFM) further confirmed the presence of highly conductive regions corresponding to the aggregated PBFDO phases (Figure S5, Supporting Information).

The intrinsically high softness of hydrogel enables intimate conformal contact with biological tissues (Figure 1c), making it suitable for bio‐adhesive or implantable electronic devices. PAIh can be applied to fabricate OECTs serving as the functional layer for monitoring various physiological signals (e.g., ECG and EOG), disease‐related signals (e.g., nociceptive potentials), and biomolecular targets (e.g., glucose) (Figure 1b,e).

### 2.2 Electrical and Mechanical Properties of PAIh Semiconductor Hydrogels

**Figure 2:**
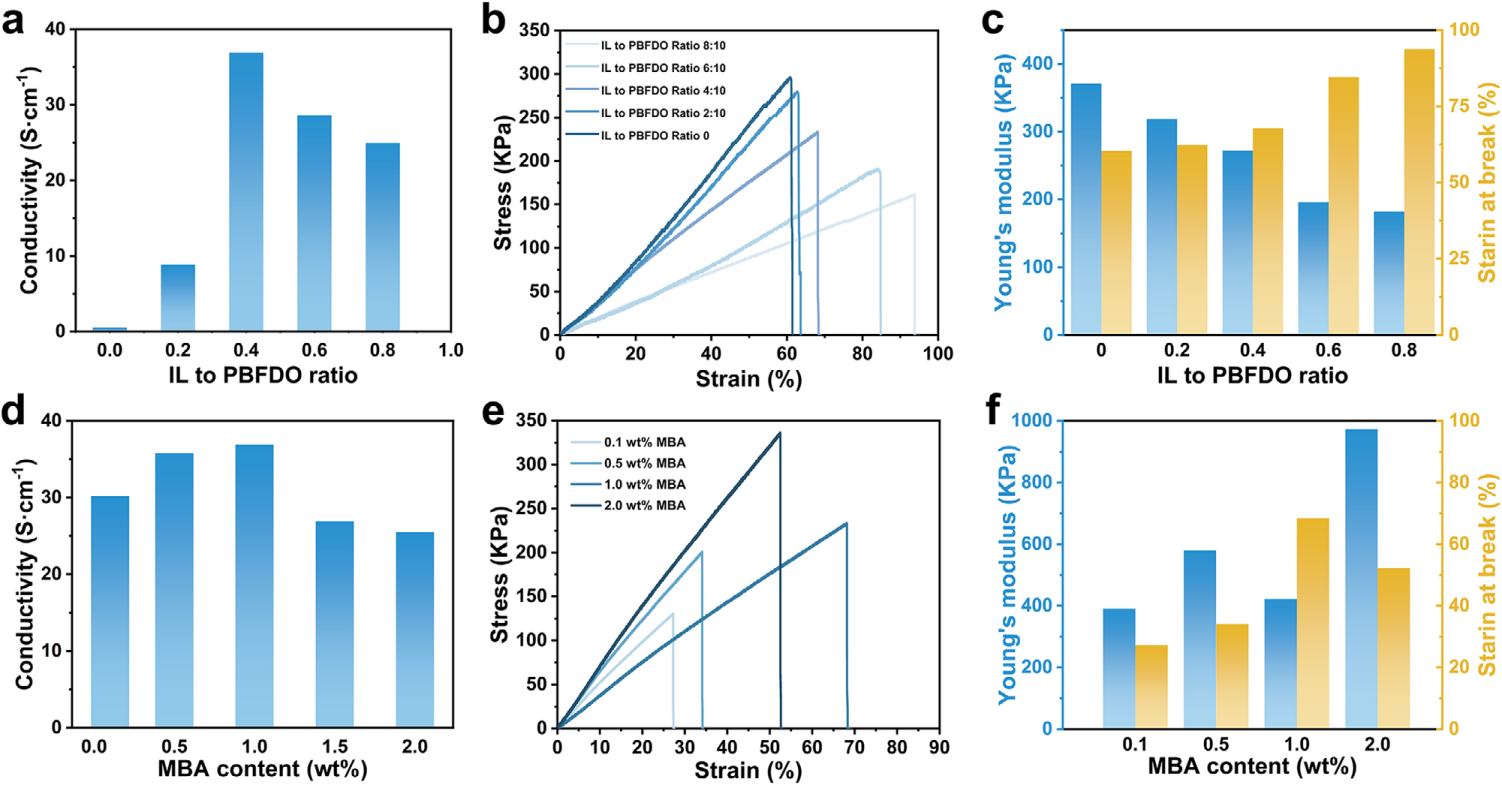
Conductivity of PAIh with varying a) IL and d) MBA content. Stress–strain curves of PAIh with varying b) IL and e) MBA content, and corresponding Young's modulus and break strains for c) IL and f) MBA content.

The performance failure of implantable bioelectronic devices often stems from mechanical mismatch and signal distortion at the interface between biological tissues and electronic components.^[^ ^28^ ^]^ To address these challenges, the electrical and mechanical properties of PAI hydrogels can be precisely tuned by modulating the concentrations of the IL and the cross‐linker MBA, enabling them to meet the stringent requirements of implantable bioelectronic devices. Although pure PBFDO inherently exhibits high conductivity, its conductivity drastically decreases to 0.07 S cm^−1^ upon gelation in the absence of IL (**Figure** **2**a). This reduction results from the formation of the PAAm cross‐linked network, which disrupts conductive pathways and increases the inter‐domain distance between conductive regions. With a fixed PBFDO:AAm mass ratio of 1:20, the introduction of IL markedly enhances conductivity. At an IL to PBFDO mass ratio of 0.2, conductivity rises by approximately two orders of magnitude to 8.8 S cm^−1^, demonstrating that even a small amount of IL can significantly improve the semiconductor hydrogel's electrical performance. The maximum conductivity of 36.9 S cm^−1^ is achieved at an IL:PBFDO mass ratio of 0.4. This enhancement arises because IL increases polymer chain mobility, which facilitates the rearrangement and tighter packing of PBFDO chains or domains.^[^ ^27^ ^]^ Consequently, phase separation between the conductive PBFDO and insulating PAAm occurs, forming interconnected clusters or channels that reduce the average inter‐domain distance and ensure continuous electron transport. However, further increases in IL content led to more pronounced phase separation, reduced gel homogeneity, and partial disruption of conductive pathways, resulting in decreased conductivity. The PAIh hydrogel, which initially exhibits a modulus of 360 kPa and a 61% fracture strain in the absence of IL, becomes progressively softer and more deformable as IL content increases (Figure 2b, c). This trend, characterized by a decreasing modulus and rising fracture strain, can be attributed to the plasticizing effect of IL, which effectively softens the hydrogel network.

The concentration of MBA directly determines the cross‐linking density of PAIh hydrogels. At low MBA concentrations, the conductivity of the PAIh is slightly enhanced. whereas at 1.5 wt.% MBA, excessive cross‐linking reduces conductivity (Figure 2d). This could be attributed to the fact that a moderate increase in cross‐linking density reduces swelling, which facilitates charge transport, whereas excessive MBA content introduces more insulating moieties. The mechanical properties are also critically governed by the MBA concentration, which dictates the cross‐linking density of the hydrogel network (Figure 2e,f). At low concentrations, insufficient cross‐linking combined with phase separation results in a loose and fragile gel with a fracture strain of only 30%. By optimizing the concentration to 1 wt.%, the network integrity is substantially improved, boosting the fracture strain to a peak of 69%. Beyond this optimal point, however, further increasing the MBA content leads to a stiffer but more brittle material with a higher modulus and lower fracture strain, indicating that cross‐linking density has become the dominant factor controlling the mechanical behavior.

The stretchability of PAIh was further evaluated by measuring resistance variations (R/R_0_) under different strains. As shown in Figure S6 (Supporting Information), PAIh were subjected to 1000 stretching cycles at 30% strain, during which they maintained stable resistance. At 50% strain, R/R_0_ increased slightly to 1.5 after 1000 cycles, indicating acceptable cyclic stretchability. In contrast, at 60% strain, the resistance increased significantly, likely due to irreversible structural damage caused by excessive stretching. These findings demonstrate that PAIh possesses highly tunable and stable electrical and mechanical properties, with softness and stretchability similar to biological tissues, thereby underscoring its strong potential for bioelectronic interface applications.

### 2.3 PAIh‐Based All‐Hydrogel OECTs

To investigate the ion‐electron coupled conduction of PAIh while meeting the amplification requirements of implantable bioelectronic devices, hydrogel OECTs were fabricated using PAIh as the semiconductor channel layer (Figure S7, Supporting Information). The device consists of a poly(acrylic acid) (PAAc) hydrogel substrate, Ag–Au NWs as flexible electrodes and an Ag/AgCl paste‐modified gate, which enables rapid and stable electrochemical doping. A NaPF_6_/PAAm hydrogel serves as the electrolyte between the gate and the semiconductor channel. Conventional Cr/Au electrodes, commonly used on rigid substrates, are inherently hard and incompatible with soft hydrogel substrates. Ag–Au NWs were prepared to form a dense conductive network capable of conformally coating hydrogel surfaces while maintaining stable conductivity under bending (Figures S8 and S9, Supporting Information). The Au shell ensures stable electrode interfaces during OECT operation, and the nanowires electrodes are compatible with photolithography‐assisted patterning, enabling well‐defined electrode geometries (Figure S1, Supporting Informationb). Figure S10 (Supporting Information) outlines the fabrication process of the hydrogel OECT, including photopatterning, transfer of Ag–Au NWs integrated with PAIh hydrogels, and the gate electrode was modified with Ag/AgCl paste via a precise dispensing technique. Given the controllable patterning technique, the fabrication of hydrogel OECT arrays was also demonstrated (Figure S17, Supporting Information). For device assembly, the NaPF_6_/PAAm precursor solution was deposited between the gate and the channel, followed by UV‐induced gelation to form the electrolyte layer.

**Figure 3:**
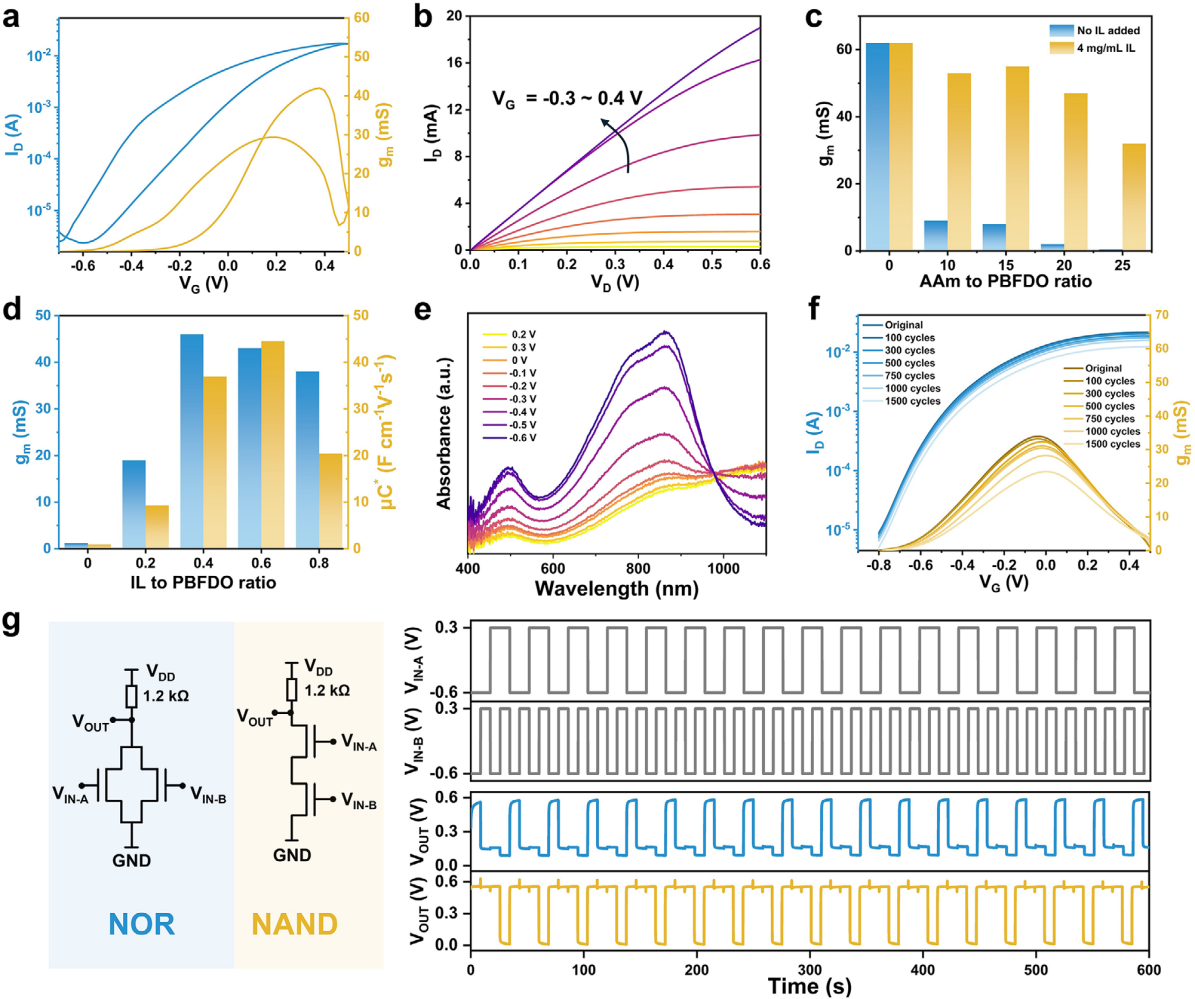
a) Transfer characteristics and transconductance (*V*_D_ = 0.6 V) and b) output characteristics curves of hydrogel OECTs based on PAIh. The dimensions of these devices were set to *W* = 1000 µm, *L* = 100 µm and *d* = 4.5 ± 0.8 µm. c) Effect of IL on the transconductance of PAIh‐based hydrogel OECTs with varying AAm content. d) Effect of PAIh with varying IL‐to‐PBFDO mass ratios on hydrogel OECT performance, characterized by transconductance and µC^*^. e) Normalized in situ electrochemical UV–vis spectra of PAIh, with an IL‐to‐PBFDO ratio of 0.4, as *V*_G_ is varied from 0.2 to −0.7 V. f) Bending cyclic stability of the hydrogel OECT device at a 5 mm bending radius. g) Construction of NAND and NOR logic circuits based on hydrogel OECTs, with circuit connections shown on the left and digital signal processing tests on the right.

Device performance was evaluated by applying a gate voltage (*V*_G_), which induces ion migration into and out of the channel. These migrating ions control the electrochemical doping and dedoping of the semiconductor channel, thereby modulating its conductivity. The drain current (*I*_D_) was measured under a constant drain voltage (*V*_D_) while varying *V*_G_ to characterize this behavior. Due to the high conductivity of PBFDO, the OECT operates in depletion mode. **Figure** **3**a,b presents typical transfer and output characteristics of the hydrogel OECT. Without a gate voltage, the channel remains in the ON state. Applying a negative *V_G_* rapidly decreases channel conductivity, switching the device OFF. The ON/OFF current ratio (*I*_ON_/*I*_OFF_) reaches 10⁴, with a maximum drain current of 16 mA and a peak transconductance (g_m_) of 43 mS. The NaPF_6_‐based electrolyte enables stable operation in aqueous environments (Figure S12, Supporting Information). Compared with conventional NaCl‐based electrolytes, the NaPF_6_‐based electrolyte exhibits superior cycling stability (Figure S12b, Supporting Information). This is due to the weaker hydration of PF_6_^−^, which mitigates irreversible water‐induced structural effects on PBFDO during doping.^[^ ^25^ ^]^ Furthermore, the hydrogel structure enhances the device's operational speed. When compared to a pure PBFDO film (τ_on_ = 276 ms) under a −0.6 V gate voltage pulse, the PAIh hydrogel‐based device demonstrated a faster response time of 188 ms (Figure S14, Supporting Information). This enhancement is attributed to the porous structure of the PAIh hydrogel, which facilitates electrolyte penetration into the semiconductor volume and accelerates the electrochemical doping process.^[^ ^17^ ^]^

To clarify the role of IL in device performance, the transconductance of OECTs fabricated with varying AAm concentrations was compared with and without IL. In the absence of IL, the transconductance of hydrogel‐based OECTs declined sharply and continued to decrease with increasing AAm concentration. When the AAm‐to‐PBFDO ratio reached 25, the devices nearly lost their semiconducting functionality. By contrast, with IL incorporation (IL‐to‐PBFDO mass ratio fixed at 0.4), the devices maintained high transconductance values exceeding 30 mS across all tested AAm concentrations (Figure 3c). The effect of IL concentration was further examined by fixing the AAm concentration at 200 mg mL^−1^ while varying IL content. As shown in Figure 3d, the transconductance first increased and then decreased with rising IL levels, reaching a maximum of 42 mS at an IL‐to‐PBFDO ratio of 0.4. The product of mobility and capacitance (µC^*^) followed the same trend. This indicates that IL can greatly enhance the semiconductor electron‐ion coupling transport performance of PAIh at a certain range of concentrations.

The electrochemical doping and dedoping processes of PAIh during OECT operation were investigated using in situ electrochemical UV–vis absorbance (Figure 3e). Starting from a high‐conductivity state, increasingly negative *V*_G_ (up to −0.6 V) induced two distinct absorption bands: a high‐energy band at 468 nm (*π*–*π* ^*^ transitions) and a broad band ≈850 nm (indicative of intramolecular charge transfer). These spectral signatures are consistent with those of pure PBFDO films, confirming that PAIh retains the intrinsic electrochemical characteristics of its semiconducting backbone. The potential of hydrogel OECTs for logic circuit implementation was then explored. As illustrated in Figure 3g, two depletion‐mode OECTs connected in parallel and then connected in series with a resistor were configured as a NOR, and two depletion‐mode OECTs connected in series with a resistor were configured as a NAND. Both logic gates exhibited stable switching behavior under different input voltage combinations (*V*_IN‐A_ and *V*_IN‐B_), thereby demonstrating the feasibility of constructing all‐gel circuits with advanced digital computing capability. Finally, the mechanical stability of the devices under deformation was evaluated. At a bending radius as small as 5 mm, the maximum *I*_D_ retained 87% of its initial value, and the transconductance remained at 85% (Figure S16, Supporting Information). After 1000 bending/recovery cycles at the same radius, the transconductance was still maintained at 80% (Figure 3f), highlighting excellent flexibility and resilience. This robustness is attributed to the intrinsic stretchability of PAIh and the bending tolerance of the Ag–Au NWs electrodes, which together enable conformal integration with soft biological tissues while maintaining the sensitivity and stability of subsequent implantable signal acquisition devices.

### 2.4 In Vitro and In Vivo Biocompatibility

**Figure 4:**
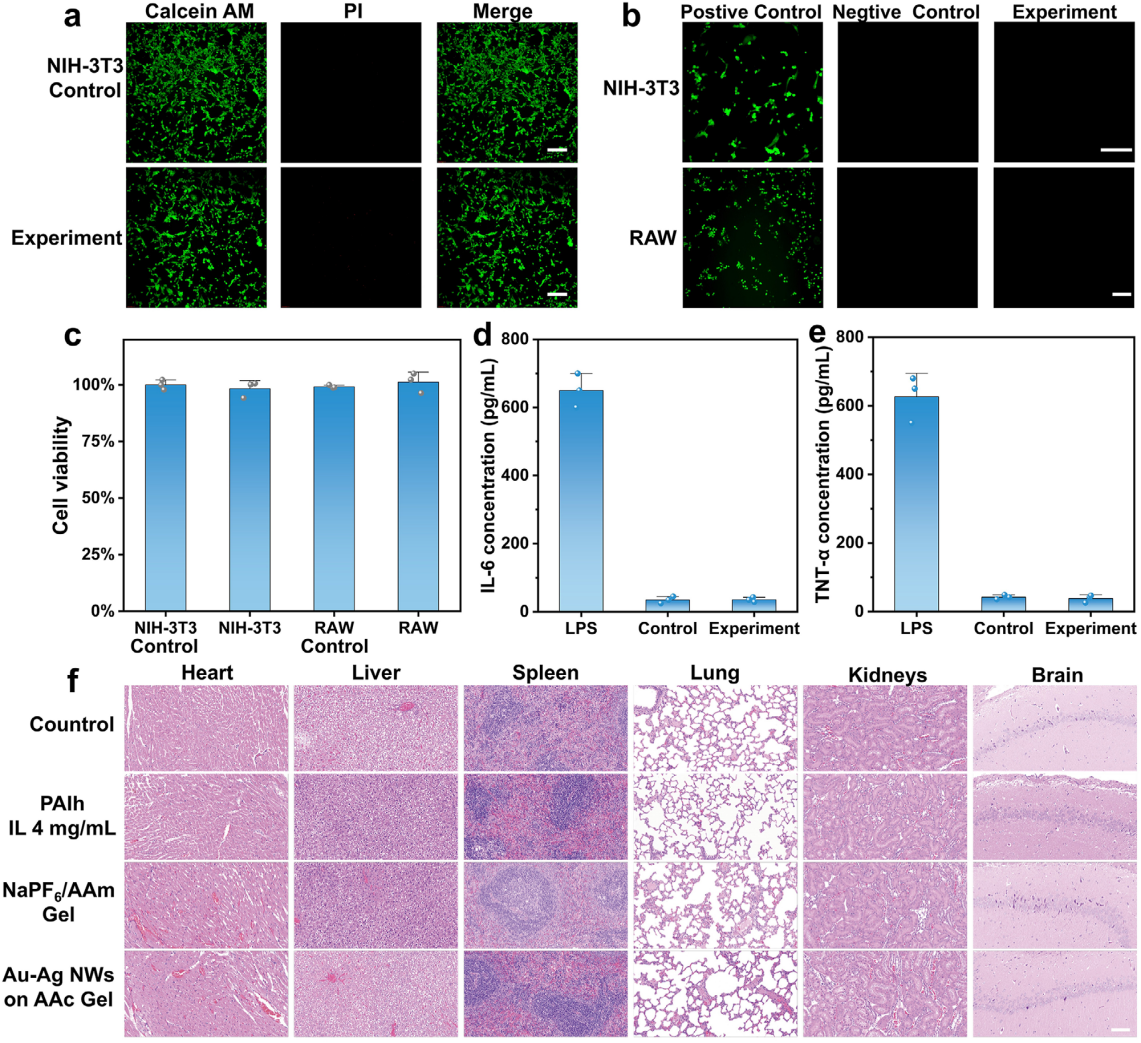
a) Live/dead stain after 72 h of culture of NIH‐3T3 cells using hydrogel OECT extracts (green fluorescence represents a live cell, and red fluorescence represents a dead cell). b) Detection of intracellular ROS in NIH‐3T3 and RAW264.7 cells after 24 h treatment with hydrogel OECT extracts. c) Cell viability after 72 h of culture of NIH‐3T3 cells and RAW264.7 cells using OECT extracts. Detection of d) IL‐6 and e) TNF‐α levels in the culture medium of RAW264.7 cells treated with hydrogel OECT extracts. f) H&E‐stained images of the heart, liver, spleen, lung, kidneys, and brain tissues collected 4 weeks after subcutaneous implantation of OECT devices in rats, including all structural components such as PAIh, Ag–Au NWs electrodes, and NaPF_6_/PAAm hydrogel electrolyte. All scale bar = 100 µm.

To evaluate the biocompatibility of the hydrogel OECTs, we first performed in vitro cell viability assays using NIH‐3T3 fibroblasts and RAW264.7 macrophages (**Figure** **4**a; Figure S18, Supporting Information). Cell Counting Kit‐8 (CCK‐8) assays demonstrated that NIH‐3T3 fibroblasts and RAW264.7 macrophages exposed to OECT extracts exhibited excellent cell viability, with survival rates exceeding 99% (Figure 4c). Consistently, Live/Dead staining revealed predominant green fluorescence and negligible red fluorescence, indicating healthy cell morphology and absence of apoptosis.

Enzyme‐linked Immunosorbent Assay (ELISA) analysis further confirmed that levels of proinflammatory cytokine (IL‐6 and TNF‐α) in the OECT‐treated group were significantly lower than those in LPS‐stimulated controls (*p* < 0.05), suggesting that the material did not trigger inflammatory cytokine production (Figure 4d,e). In parallel, intracellular Reactive Oxygen Species (ROS) detection showed that, unlike the strong fluorescence observed in the H_2_O_2_‐treated positive control, cells exposed to OECT extracts displayed no excessive ROS accumulation, highlighting the absence of oxidative stress (Figure 4b). Together, these results support the in vitro biosafety of the OECT devices.

To evaluate host responses in vivo, the individual components of the OECT were implanted subcutaneously in rats for 4 weeks. Histological examination of major organs (heart, liver, spleen, lungs, kidneys, brain, and skin) revealed normal architecture without evidence of tissue injury or inflammatory infiltration. At the implantation site, both H&E and Masson's trichrome staining showed no obvious inflammatory cell infiltration, and collagen deposition appeared within normal range, indicating minimal local tissue reaction (Figure 4f; Figure S19, Supporting Information).

Overall, both in vitro and in vivo results consistently demonstrated that the hydrogel OECT possesses excellent biocompatibility and does not elicit cytotoxicity, inflammatory responses, or oxidative stress, thereby supporting its potential for safe biomedical applications.

### 2.5 Biosensing and Implantable Physiological Signal Monitoring

Building on the superior mixed ionic–electronic conduction, high transconductance, low modulus, and excellent biocompatibility of the hydrogel OECTs described above, their potential was further explored in highly sensitive biomolecular sensing (glucose detection), and in recording and amplifying weak physiological signals (ECG and EOG), thereby enabling applications in health monitoring and medical diagnostics.

**Figure 5:**
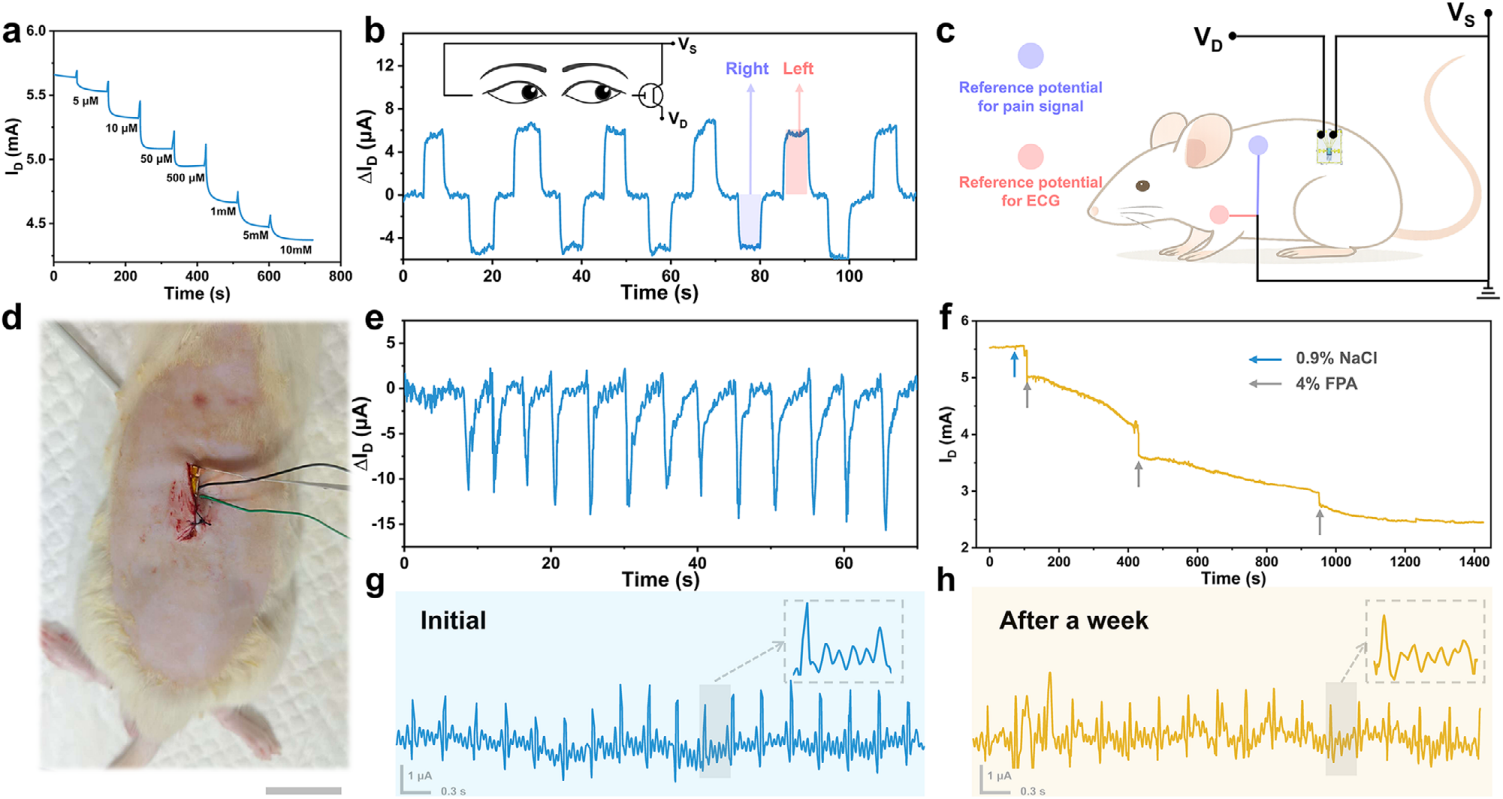
a) Glucose sensing performance based on hydrogel OECTs. b) EOG signals recorded using a hydrogel OECT during horizontal eye movements, and the inset shows the circuit connection. c) Schematic illustration of a subcutaneously implanted hydrogel OECT device in the back for monitoring physiological signals in rats. For measurements of potential variations corresponding to nociceptive stimuli, the reference electrode was placed the upper back. For ECG measurements, it was placed on the upper abdomen. d) Photograph of the implantable hydrogel OECT subcutaneously implanted under the dorsal region of a rat for physiological signal monitoring, scale bar: 2 cm. e) I_D_ variations induced by back nociceptive stimuli potentials triggered by intermittent mechanical clamping of the rat hind limb (at 5 s intervals). f) I_D_ variations after subcutaneous injection of saline and PFA in rats. g) Subcutaneously implanted device monitoring the rat's ECG signals initially and h) after one week. All devices were tested at a *V*_D_ of 0.6 V.

An electrochemical glucose biosensor was developed based on the hydrogel OECT, in which glucose oxidase was immobilized in the channel via glutaraldehyde cross‐linking. Upon the introduction of glucose into the electrolyte, an enzymatic cascade generated H_2_O_2_, which was subsequently oxidized to gluconolactone with concomitant electron transfer, which in turn modulated the channel conductivity. As shown in **Figure** **5**a, the *I*_D_ gradually decreased with increasing glucose concentration from 10^-2^ to 10 mM. Because bioelectrical signals typically have amplitudes ranging from microvolts to millivolts, the amplification capability of OECTs renders them particularly suitable for accurate recording and analysis. The capability of the hydrogel OECT to record human EOG and ECG signals was first demonstrated. As illustrated in Figure 5b, with the gate electrode attached to the right side of the eye and the source electrode connected to the left side, eye movements were successfully monitored by recording I_D_ under a drain bias of 0.4 V, exhibiting a peak current amplitude of ≈12 µA across distinct signal patterns corresponding to leftward, central, and rightward positions. The capability of the OECT to capture and amplify ECG signals was also evaluated (Figure S20, Supporting Information).

For implantable applications, a flexible device was designed, in which OECT electrodes were connected to external copper wires using silver paste, and the source, drain, and silver‐paste junctions were encapsulated with polyimide films, thereby preventing signal interference and silver leakage. The device was subcutaneously implanted into the back of rats to enable in vivo electrophysiological signal recording (Figure 5c,d). In one experiment, intermittent mechanical pinching of the rat hind limb (every 5 s) was applied to induce acute nociceptive stimulation, which led to local ionic and potential fluctuations in the dorsal tissue. The implanted OECT was able to record these neural regulation‐induced potential variations in real‐time (Figure 5e), with I_D_ fluctuations of 10–15 µA. A murine model of neurogenic inflammatory pain was further established through subcutaneous injection of 4% paraformaldehyde (PFA, 100 µL per injection). The implanted OECT was used to monitor resting potential changes in the back tissue associated with pain‐induced local inflammation (Figure 5f). Each PFA injection produced discernible variations in tissue potential, which were reflected as changes in I_D_.

Finally, the long‐term implantability of the device for monitoring ECG signals in rats was evaluated. Immediately after implantation, the device reliably recorded cardiac activity with peak amplitudes of ≈3 µA, thereby demonstrating sufficient sensitivity to weak bioelectric signals (Figure 5g). Importantly, the device maintained stable operation for at least 1 week post‐implantation, with the average R‐wave amplitude of ECG signals retaining more than 85% of its initial value and exhibiting no noticeable baseline drift or signal distortion (Figure 5h). These results demonstrate the long‐term stability and reliability of the hydrogel OECTs for continuous physiological monitoring in vivo.

## 3 Conclusion

In this work, we developed a novel n‐type depletion mode semiconductor hydrogel through IL‐mediated phase separation, which facilitated the formation of interconnected conductive networks of PBFDO. The resulting hydrogel combines high intrinsic conductivity, tissue‐like softness, and efficient coupled ionic‐electronic transport. All‐hydrogel OECTs fabricated from this material exhibit excellent electrical performance, achieving a maximum transconductance of 43 mS, as well as mechanical flexibility with stable operation over 1500 bending cycles. The low‐modulus structure also ensures good biocompatibility, with negligible cytotoxicity and no obvious inflammatory response, enabling safe integration with biological tissues. Furthermore, these devices support real‐time monitoring of human ECG and EOG signals via surface attachment, as well as subcutaneous recording of nociceptive stimuli and ECG signals in rats, maintaining stable operation in vivo for 1 week. This study establishes a robust materials and device strategy for multi‐parameter physiological signal monitoring and provides a promising platform for pain assessment, cardiovascular health monitoring, and precision closed‐loop therapy. Overall, the design concept of all‐hydrogel electronics introduced here offers a critical framework for building more advanced bio‐integrated diagnostic and therapeutic systems, demonstrating a transformative potential for future clinical applications and health management.

## 4 Experimental Section

### Materials

All chemicals and raw materials were obtained from commercial suppliers and used without further purification. PBFDO (10–12 mg mL^−1^ in DMSO) was purchased from Volt‐Amp Optoelectronics Tech. Co., Ltd., Dongguan, China. Acrylamide (AAm, ≥ 99%) and N,N′‐methylenebisacrylamide (MBA, ≥ 99%) were obtained from Aladdin. Photoinitiators 2‐hydroxy‐2‐methylpropiophenone (Irgacure 1173, 98%) and 2‐hydroxy‐4′‐(2‐hydroxyethoxy)‐2‐methylpropiophenone (Irgacure 2959, 98%), ionic liquid 1‐ethyl‐3‐methylimidazolium bis(trifluoromethylsulfonyl)imide ([EMIM]+ [TFSI]‐, ≥98%), acrylic acid (AAc, 98%), and glycerol (AR, ≥ 99%) were obtained from Shanghai Titan Scientific Co., Ltd. Poly(ethylene glycol) diacrylate (PEGDA, average M_n_ = 575 Da) was obtained from Sigma‐Aldrich. ROL‐7133 photoresist was obtained from Xian Boyan Micro‐nano Informational Technology Co., Ltd.

### Preparation and Patterning of Semiconductor Hydrogel (PAIh) Films

PAIh films were prepared in a nitrogen‐filled glovebox. To prepare the precursor solution, AAm was added to a commercial PBFDO solution (10 mg mL^−1^ in DMSO) to achieve an AAm concentration of 200 mg mL^−1^. MBA and the photoinitiator 1173 were subsequently added at 1 wt.% relative to AAm. Ionic liquid [EMIM]^+^ [TFSI]^−^ (IL) was incorporated at a mass ratio of IL:PBFDO = 0.4, unless otherwise specified. The resulting precursor solution was stirred overnight to ensure complete mixing. For hydrogel film formation, silicon or glass substrates were plasma‐treated for 10 min prior to spin‐coating. The PAIh precursor solution was spin‐coated at 3000 rpm for 30 s, followed by UV irradiation (365 nm, 40 mW cm^−^^2^) for 10 min to initiate gelation. Then, the films were removed from the glovebox and immersed in DI water for 1 min to yield the PAIh films. The typical thickness of the PAIh film is ≈4.5 ± 0.8 µm.

For patterning, the precursor solution was spin‐coated onto silicon substrates. A photomask defining the desired pattern was placed on top, and the sample was exposed to a 365 nm UV lamp (40 mW cm^−^^2^) for 10 min to induce localized gelation. The partially gelled films were first developed in DMSO for 30 s, followed by immersion in DI water for 1 min to obtain patterned PAIh films.

### Characterization

For scanning electron microscopy (SEM), the PAIh films were fully swollen and then freeze‐dried. SEM images were captured using a SU8010 microscope at an operating voltage of 3 kV. For atomic force microscopy (AFM), PAIh films were directly dried and measured using a Bruker Dimension Icon AFM in tapping mode with a probe of spring constant 𝑘 = 3 N m^−1^. Raman spectra were collected using an inVia Inspect Raman Microscope in the range of 600–2000 cm^−1^, with a 532 nm excitation wavelength, 10 s exposure time, and 50% laser power. Mechanical stress–strain curves were obtained using an Instron universal testing machine at a stretching rate of 5 mm min^−1^. The square resistance of PAIh films was measured using a four‐point probe tester, from which the conductivity was calculated. Electrochemical cyclic voltammetry (CV) measurements were performed on a Gamry Interface 1010E series electrochemical workstation. In situ UV–vis spectra were measured using a NOVA2S spectrometer over a wavelength range of 400–1100 nm.

### Fabrication and Characterization of All‐Hydrogel OECTs

Electrode patterning: Ag–Au core‐shell nanowires (Ag–Au NWs) were prepared according to the previous literature.^[^ ^29^ ^]^ A 20  mg mL^−1^ Ag–Au NWs ethanol dispersion was spin‐coated on a glass substrate at 500 rpm and immediately dried on a 60 °C hot plate. This coating/drying cycle was repeated four times to form a continuous conductive film. ROL‐7133 photoresist was spin‐coated on the nanowire film at 1000 rpm, baked at 110 °C for 90 s, and exposed to 360 nm UV light at 100 mW cm^−^^2^ for 15 s under a photomask. The substrate was post‐baked at 110 °C for 90 s and developed in ZX238 developer for 70  s, followed by rinsing with DI water. The substrate was then ultrasonicated in deionized water for 15 s; unprotected nanowires detached, leaving the patterned electrodes. Residual photoresist was removed with acetone.

Preparation of PAAc hydrogel substrate and device assembly: Glycerol and DI water were mixed at a mass ratio of 2:5 to serve as the solvent for the gel precursor. AAc, PEGDA, and photoinitiator 2959 were added to this solvent to achieve concentrations of 250 mg mL^−1^ for AAc and 5 mg mL^−1^ for both MBA and photoinitiator. The solution was stirred overnight to ensure homogeneity. Gelation was induced by 365 nm UV irradiation, and the PAAc hydrogel was adhered to PET. The patterned Ag–Au NWs electrodes and pre‐patterned PAIh were then transferred onto the PAAc hydrogel surface, and the gate electrode was modified with Ag/AgCl paste via a precise dispensing technique.

Electrical characterization: OECT electrical performance, including output, transfer, and stability measurements, was conducted at room‐temperature using two Keithley 2450 source meters and an electrical probe station. For performance comparison between PAIh and pure PBFDO films, devices were fabricated on silicon substrates with a SiO_2_ insulating layer. For the bending cycle tests, to prevent drying of the hydrogel electrolyte, a small amount of water was added around the surface of the hydrogel, and additional water was supplied every 500 cycles throughout the experiment.

### Fabrication of OECTs on Silicon Substrate

The OECTs were fabricated on Si/SiO_2_ wafers treated by plasma for 10 min. Subsequently, source and drain (10 nm Cr, 100 nm Au) on the substrate were deposited by thermal evaporation and patterned by photolithography using an UV Maskless Lithography machine (TuoTuo Technology, UV LithoACA). PAIh was patterned to define the channel using the method mentioned previously. For pure PBFDO thin films, excess part is removed using a cotton swab to define the channel. Ag/AgCl pellets were used as the gate electrode for device testing.

### Cell Culture

NIH‐3T3 fibroblasts (RRID: CVCL_0594) and RAW264.7 macrophages (RRID: CVCL_0493) were obtained from the Cell Bank of the Chinese Academy of Sciences (Shanghai, China). All cell lines were tested negative for mycoplasma contamination prior to use. Cells were cultured in Dulbecco's modified Eagle's medium (DMEM, Gibco, USA) supplemented with 10% heat‐inactivated fetal bovine serum (FBS, Gibco, USA), 100 U mL^−1^ penicillin, and 100 µg mL^−1^ streptomycin. All cultures were maintained at 37 °C in a humidified atmosphere with 5% CO_2_.

### Cell Viability Assay (CCK‐8)

The cytocompatibility of the OECT device was assessed using the Cell Counting Kit‐8 (CCK‐8, Dojindo, Japan). Briefly, NIH‐3T3 and RAW264.7 cells were seeded into 96‐well plates (2 × 10⁴ cells/well) and cultured for 24 h. The medium was then replaced with fresh medium containing the test samples. After 24 and 72 h incubation, 10 µL of CCK‐8 reagent was added to each well, followed by incubation for 2 h. Absorbance at 450 nm was measured using a microplate reader (BioTek, USA), and cell viability was calculated relative to the untreated control group.

### Live/Dead Staining

To further evaluate cytocompatibility, Live/Dead staining was performed. Cells were seeded in confocal dishes (2 × 10⁵ cells/well) and incubated with the test materials for 72 h. Subsequently, cells were stained with Calcein‐AM (2 µm) and propidium iodide (PI, 4 µm) for 30 min at 37 °C in the dark. After washing three times with PBS, images were acquired using an inverted fluorescence microscope (Leica, Germany). The proportion of viable (green) and dead (red) cells was analyzed to assess cell survival.

### Intracellular Reactive Oxygen Species (ROS) Assay

Intracellular ROS levels were quantified using the DCFH‐DA fluorescent probe (Beyotime, China). After treatment with the test samples for 24 h, NIH‐3T3 and RAW264.7 cells were incubated with 10 µm DCFH‐DA at 37 °C for 30 min in the dark. Excess dye was removed by washing three times with PBS. Fluorescence images were captured using a confocal laser scanning microscope (Zeiss LSM880, Germany), with excitation at 488 nm. The fluorescence intensity of DCF was quantified to evaluate intracellular ROS levels.

### Enzyme‐linked Immunosorbent Assay (ELISA)

RAW264.7 macrophages were seeded into six‐well plates and cultured overnight for adherence. Cells were then stimulated with lipopolysaccharide (LPS, 10 µg mL^−1^) or treated with OECT extract solution for 24 h. After incubation, the culture medium was collected and centrifuged at 12 000 g for 10 min at 4 °C to remove cell debris. The supernatant was harvested and analyzed using mouse IL‐6 (Proteintech, KE10007) and TNF‐α (Proteintech, KE10002) ELISA kits, according to the manufacturers’ instructions. The absorbance at 450 nm was measured with a microplate reader (BioTek, USA), and cytokine concentrations were calculated based on the standard curves.

### In Vivo Biocompatibility Assessment

Male Sprague–Dawley rats (6–8 weeks, 180–200 g, pathogen‐free) were purchased from Shanghai Jie Si Jie Experimental Animal Co., Ltd. (Shanghai, China). Animals were housed under standard conditions with a 12 h light/dark cycle. All experimental procedures were reviewed and approved by the Institutional Animal Care and Use Committee of Shanghai Jiao Tong University (approval number: A2024078).

To investigate the in vivo biocompatibility of the OECT device, subcutaneous implantation was performed in Sprague–Dawley rats. After 4 weeks, major organs (heart, liver, spleen, lungs, kidneys, and brain) were harvested and fixed in 4% paraformaldehyde. Tissues were embedded in paraffin, sectioned, and stained with hematoxylin and eosin (H&E) for histological evaluation.

At the implantation site, surrounding tissues were subjected to both H&E and Masson's trichrome staining to assess local inflammatory response and fibrous capsule formation. Histological sections were examined under a light microscope (Olympus, Japan).

### All‐Hydrogel OECTs for Glucose Sensing

Glucose oxidase (GOx) was dissolved in PBS buffer (pH 7.2–7.4) at a final enzyme concentration of 20 mg mL^−1^. Subsequently, glutaraldehyde was added to the solution to with a concentration of 5 mg mL^−1^, serving as the cross‐linking agent. The OECT devices were then immersed in the enzyme solution at room temperature and maintained for 24 h to complete the immobilization process.

### Implantable All‐Hydrogel OECTs for Physiological Signal Monitoring

The source and drain electrodes were connected to external copper wires using silver paste, and the silver‐paste junctions as well as excess portions of the source/drain electrodes were encapsulated with polyimide tape. The external wires were connected to a Gamry Interface 1010E series electrochemical workstation for voltage application and real‐time current recording. The OECT was attached to the left upper waist of a healthy adult male volunteer, with a commercial Ag/AgCl gel electrode placed on the right chest as the reference and connected to the OECT source electrode. A drain voltage of 0.4 V was applied, and current variations corresponding to ECG signals were recorded. For EOG measurements, the hydrogel OECT and a commercial Ag/AgCl gel electrode were placed on opposite sides of the human eyes in the same source/drain configuration. Written informed consent was obtained from the volunteer before participation.

For in vivo rat experiments, the device was implanted beneath the lower dorsal skin through a small incision that was sutured after insertion. Nociceptive stimuli‐related signals were recorded with a commercial Ag/AgCl electrode placed on the upper dorsal region as the reference, while ECG signals were measured with the reference electrode attached to the upper abdominal region. In both cases, a drain voltage of 0.4 V was applied, and the resulting current responses were monitored.

### Statistical Analysis

Unless otherwise stated, all data are presented as mean ± standard deviation (SD). The signal‐to‐noise ratio (SNR) was calculated as the ratio of the mean signal amplitude to the standard deviation of the baseline noise and expressed in decibels (dB), using MATLAB R2024a. Statistical analyses were performed using OriginPro 2022, and differences were considered statistically significant at *p* < 0.05.

## Conflict of Interest

The authors declare no conflict of interest.

## Supporting information



Supporting Information

## Data Availability

The data that support the findings of this study are available from the corresponding author upon reasonable request.
