# Supplementary material for: All‐Hydrogel‐Based Organic Electrochemical Transistors for Implantable Physiological Signal Monitoring
Source: Adv Sci (Weinh). 2025 Nov 14;13(16):e17375. doi: 10.1002/advs.202517375 (PMC13042953; doi:10.1002/advs.202517375)
Supplement: Supplementary file 1 — Supporting Information [file ADVS-13-e17375-s001.pdf]

Supporting Information

All-Hydrogel Based Organic Electrochemical Transistors for Implantable Physiological Signal Monitoring

Qicheng Liang†, Yue Wang†, Ruizhe Wang, Wanfang Zhang, Runcheng Hao, Yueheng Zhong, Jingling Zhang, Xiang Li, Weichu Chen, Chunyu Fan, Yuwen Zhu, Yu Sun, Hong Jiang, Hengda Sun*, and Gang Wang**

† These authors contributed equally to this work.

Q. Liang, R. Wang, W. Zhang, R. Hao, Y. Zhong, J. Zhang, X. Li, W. C. Fan, Chen, Y. Zhu, H. Sun, G. Wang

State Key Laboratory of Advanced Fiber Materials

College of Materials Science and Engineering

Donghua University

Shanghai 201620, China

E-mail: sunhengda@dhu.edu.cn ; gwf8707@dhu.edu.cn

Y. Wang, Y. Sun, H. Jiang

Department of Anesthesiology

Shanghai Ninth People's Hospital

Shanghai Jiao Tong University School of Medicine

Shanghai 200011, China

E-mail: jiangh1173@sh9hospital.org.cn

H. Sun

Henan Academy of Sciences

Zhengzhou 450046, China

Supplementary Figures

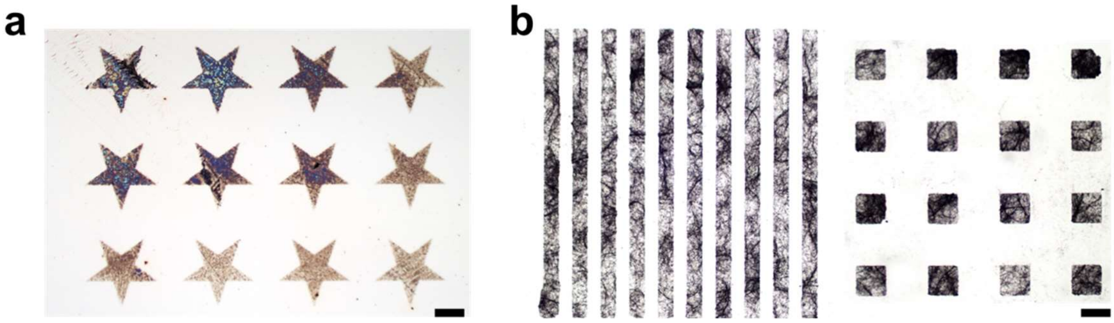

Figure S1 Patterned a) dry PAIh and b) Ag-Au NWs (scale bar, 100 μm).

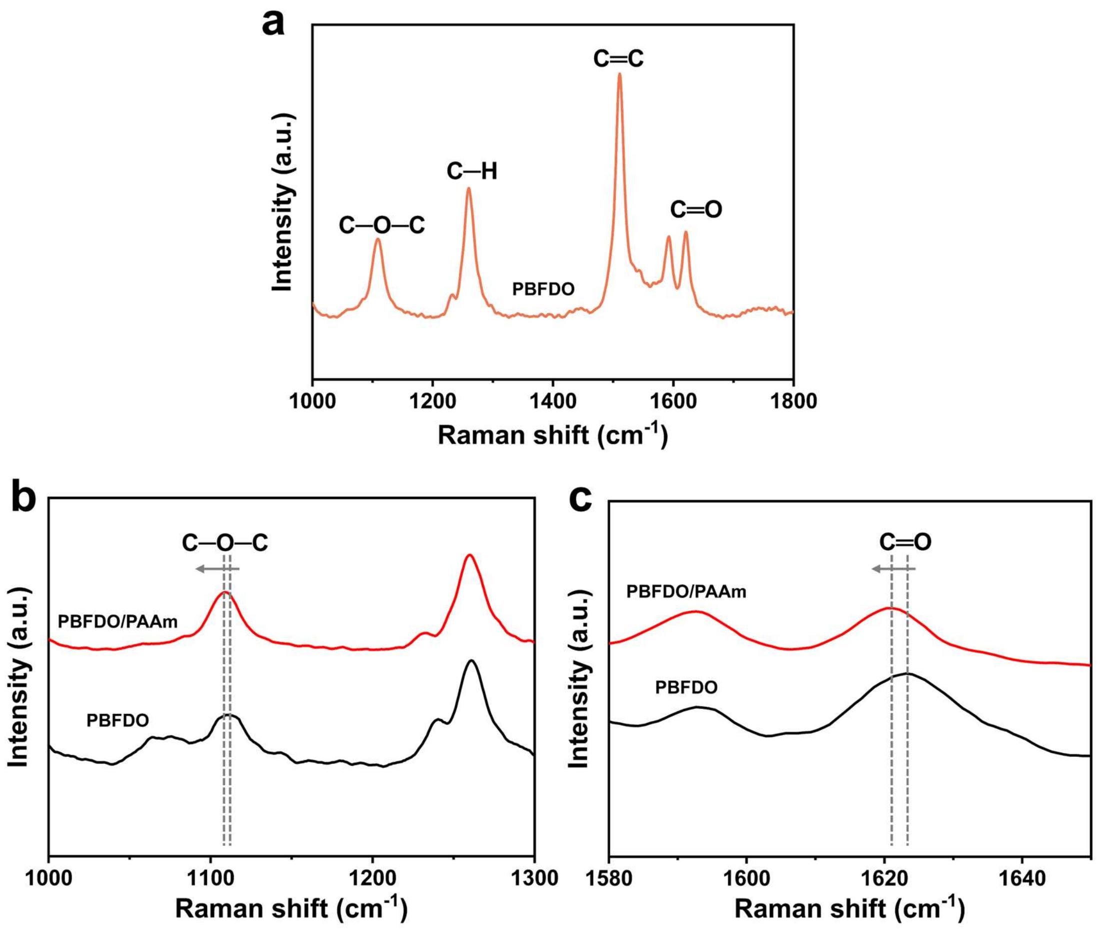

Figure S2 Raman spectra of pure PBFDO film and PBFDO/PAAm hrdrogel.

a b
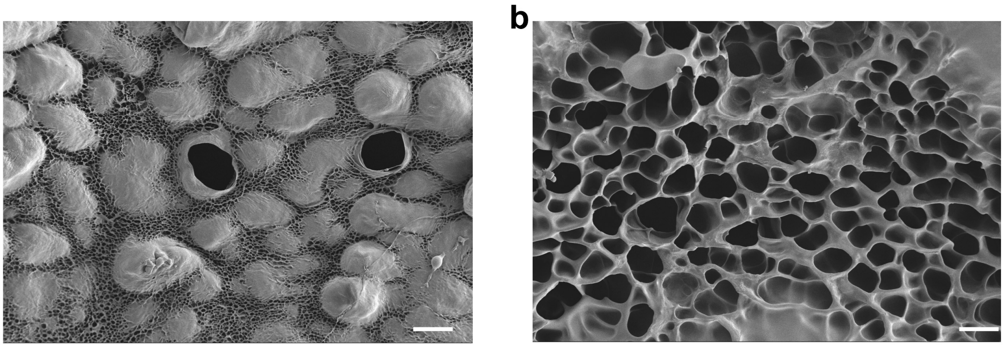

Figure S3 SEM images of freezing-dried PAIh with an IL content of 4 mg/mL, scale bar a) 100 μm and b) 10 μm.

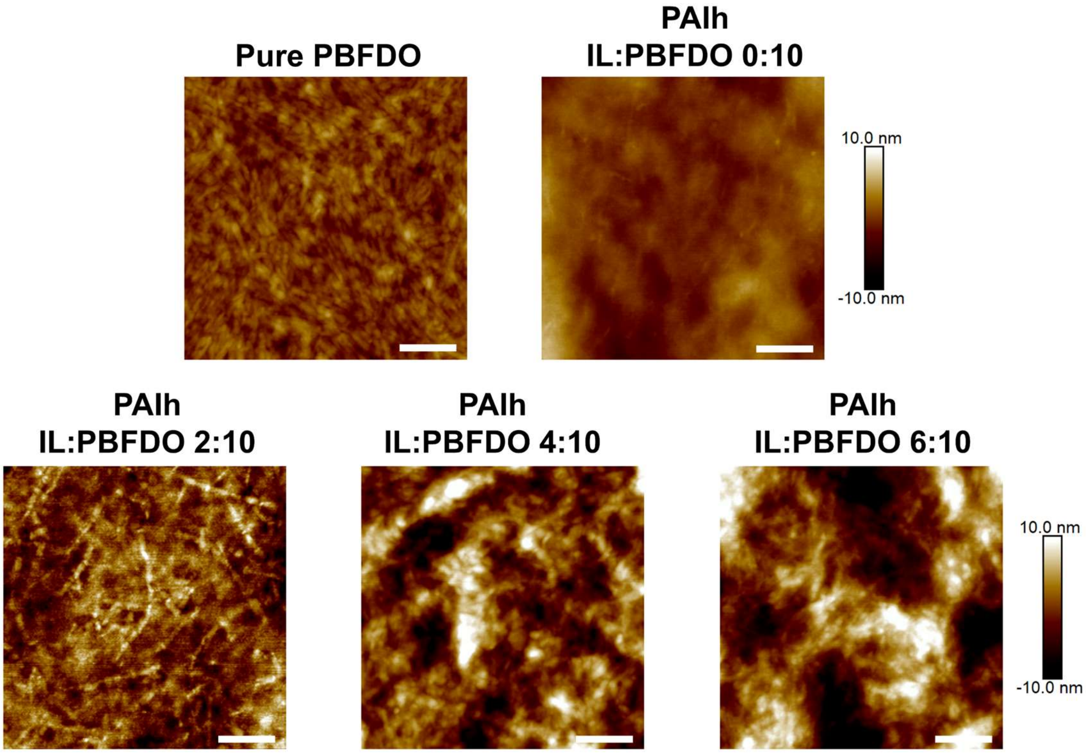

Figure S4 AFM height images of pure PBFDO film and PAIh with different IL content, scale bar 200 nm.

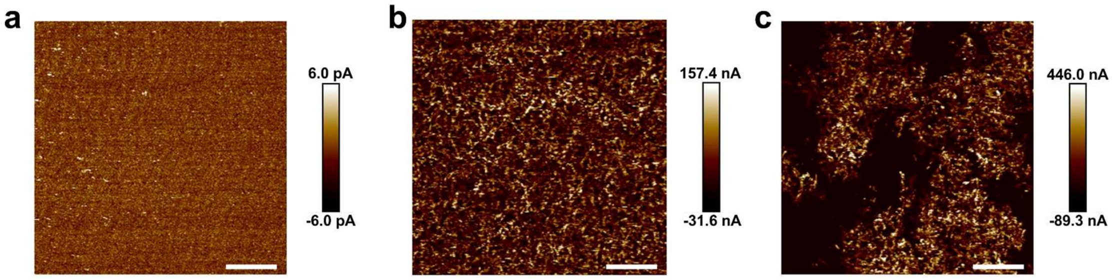

Figure S5 C-AFM images of dried PAIh films with an IL concentration of a) 0 mg mL^{-1}, applied bias of 10 V, b) 2 mg mL^{-1} and c) 4 mg mL^{-1}, applied bias of 25 mV. Scale bar 1 μm.

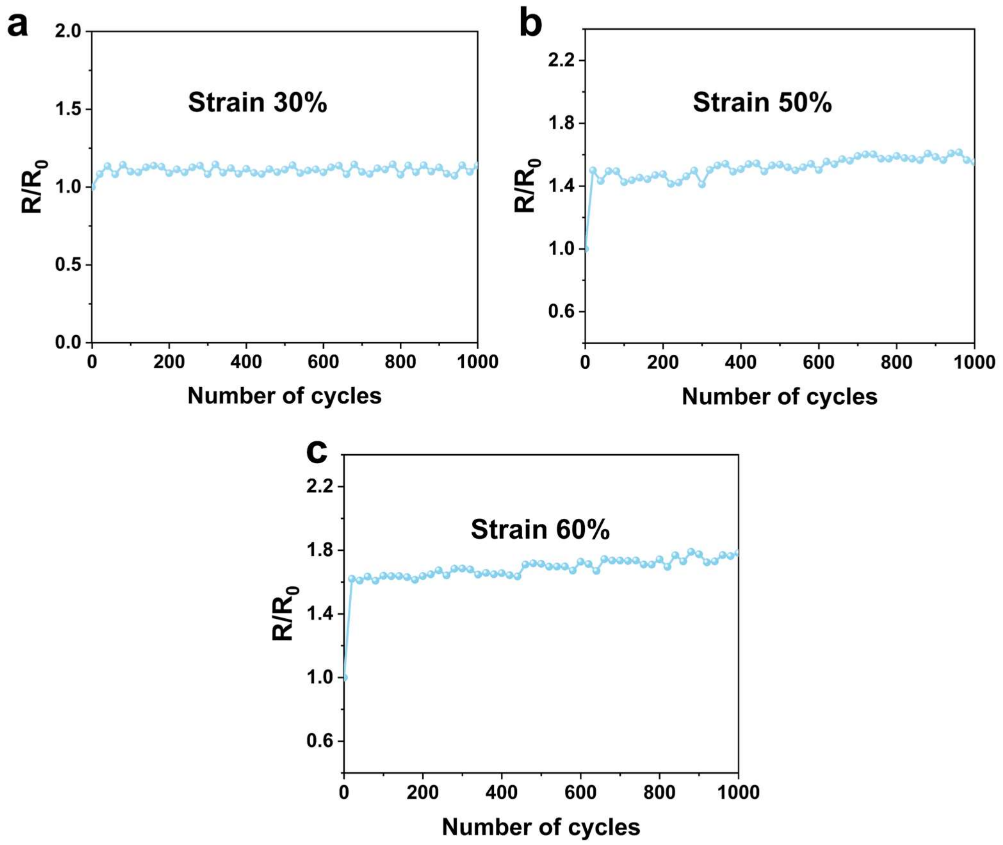

Figure S6 Relative resistance change (R/R_0) of PAIh under cyclic tensile strains of a) 30%, b) 50%, and c) 60%.

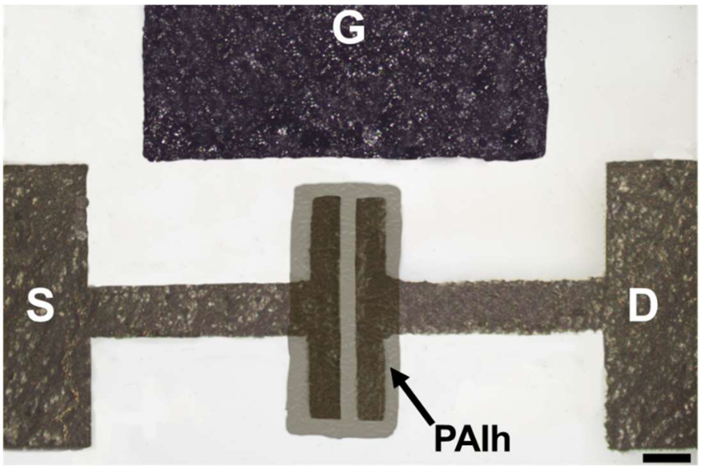

Figure S7 Hydrogel OECT devices for testing. The dimensions of device were set to W = 1000 μm and L = 100 μm. Scale bar = 200 μm.

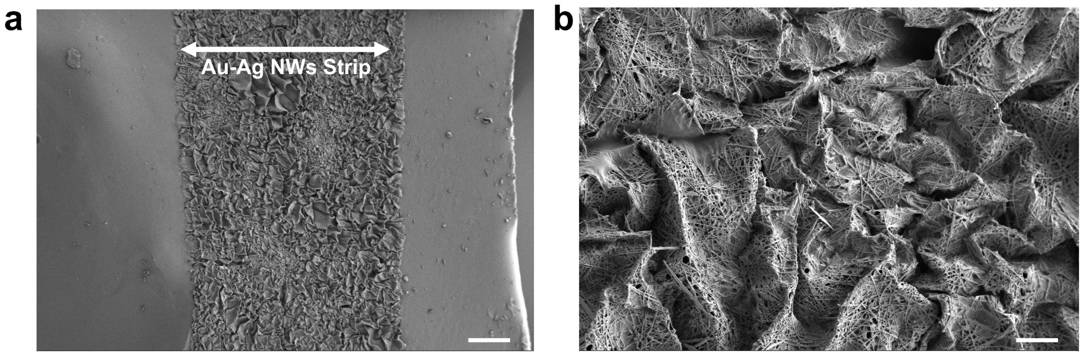

Figure S8 SEM images of a single Ag-Au NWs strip pattern attached on a PAAc hydrogel substrate, scale bar a) 100 μm, b) 10 μm.

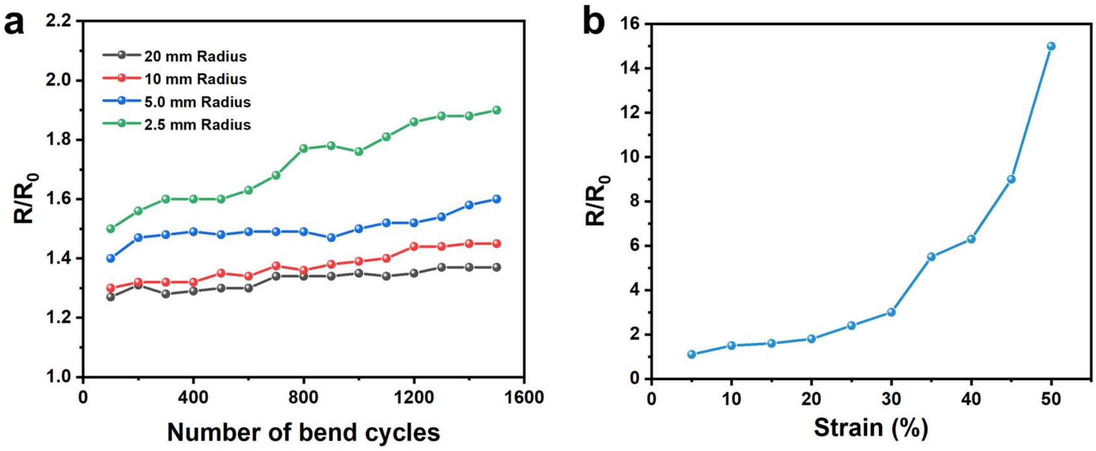

Figure S9 a) Resistance variation of Ag-Au NWs adhered to the PAAc hydrogel substrate under cyclic bending at different radii. b) Resistance variation of Ag-Au NWs during continuous stretching.

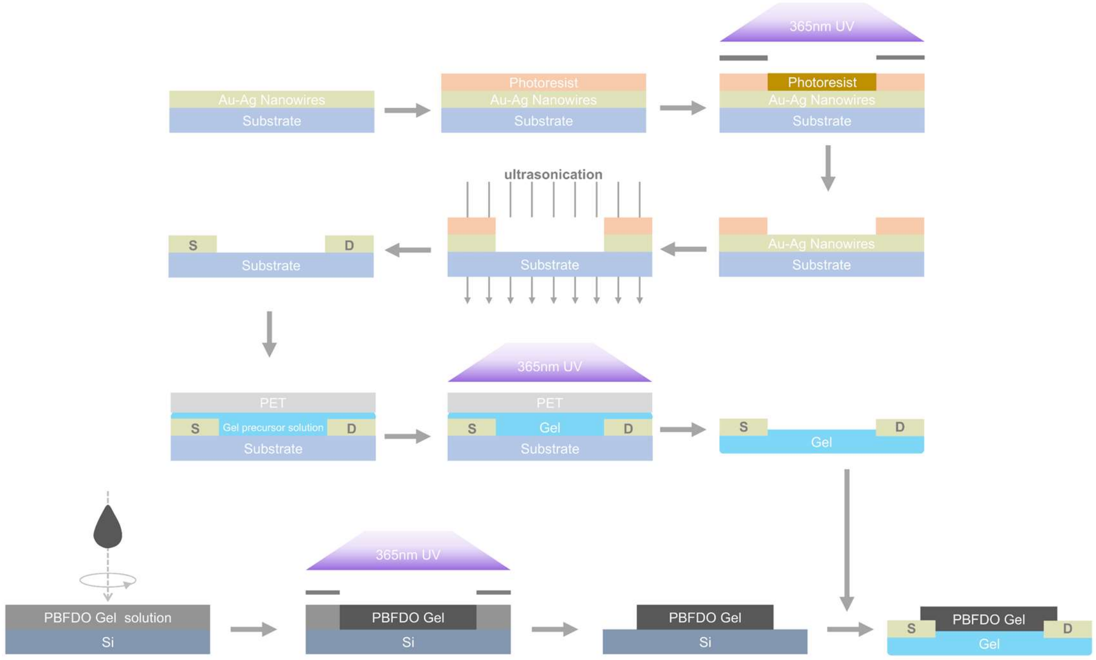

Figure S10 Schematic illustration of the fabrication procedure for the hydrogel OECT.

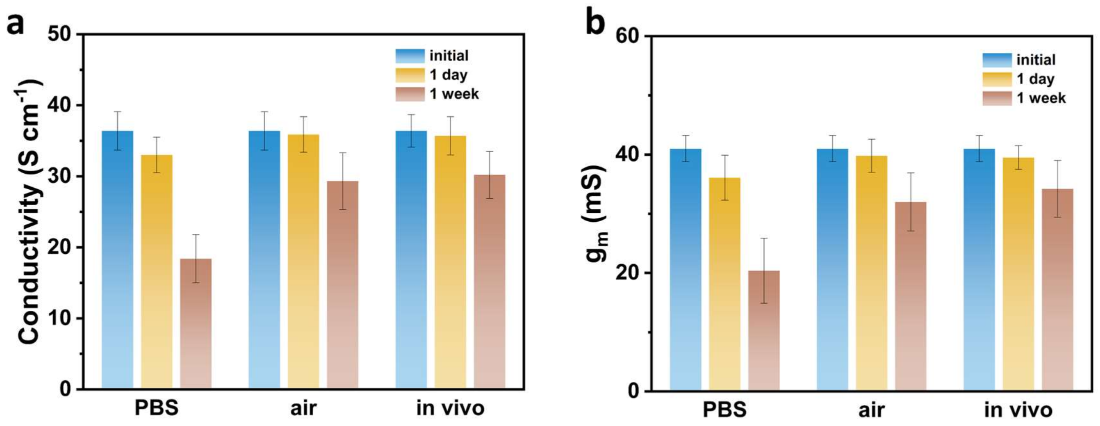

Figure S11 a) Conductivity variation of PAIh films and b) Transconductance variation of OECT devices based on PAIh after being stored for one week in PBS, air, and in vivo (subcutaneous tissue of rats).

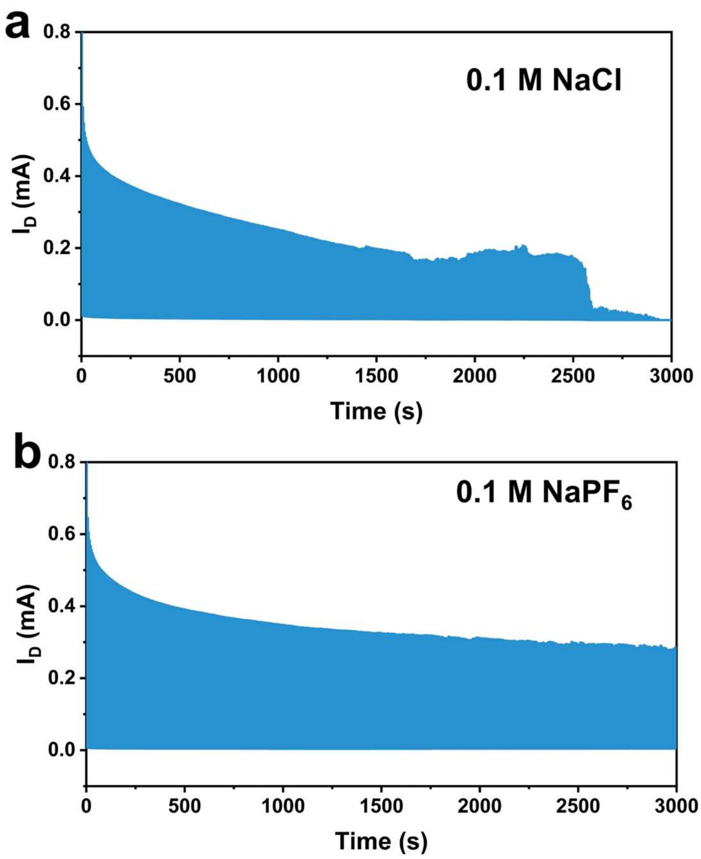

Figure S12 The cyclic stability of PAIh-based OECTs was tested using (a) 0.1 M NaCl and (b) $NaPF_6$ as electrolytes. Gate voltage pulses ranged from -0.6 V to 0 V, and 500 cycles were performed in 3000 s.

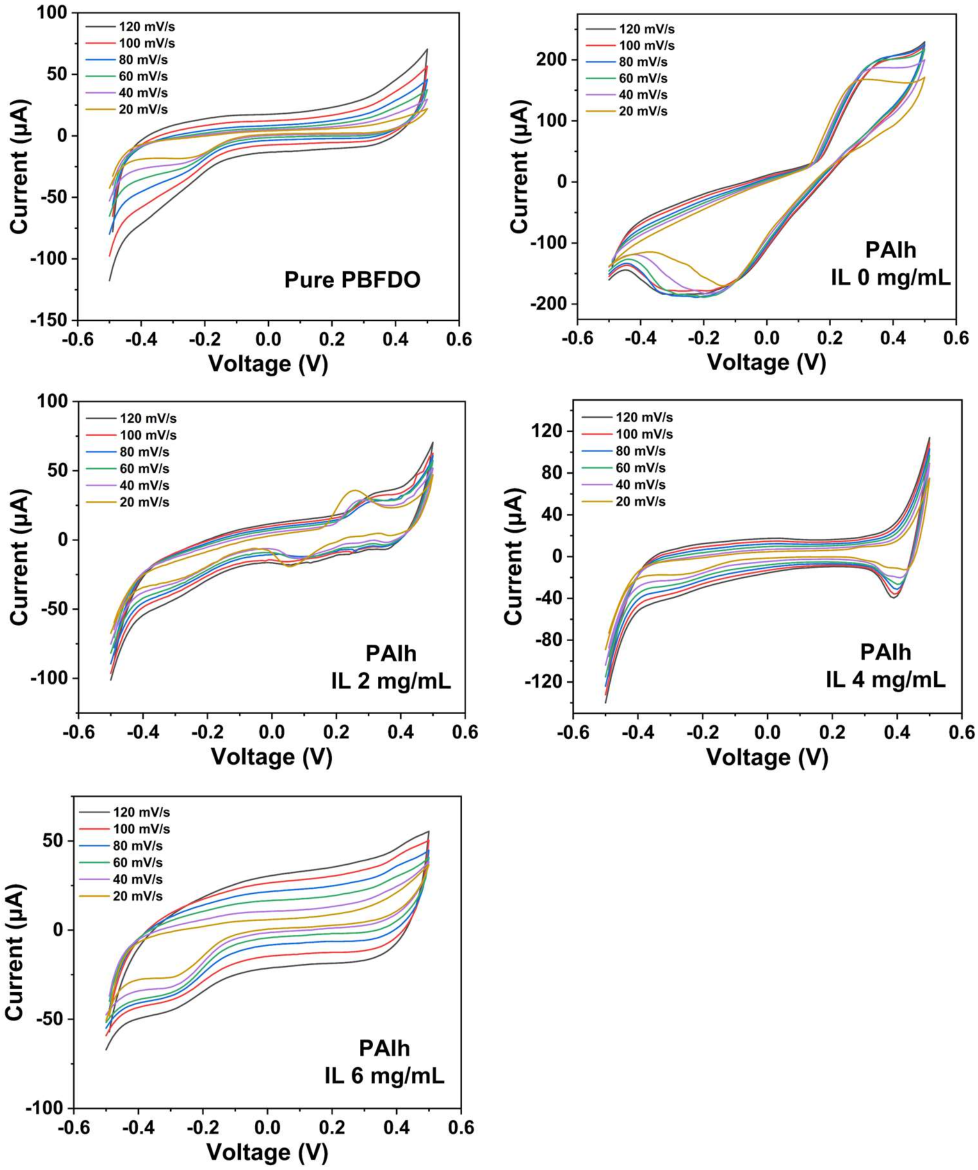

Figure S13 Cyclic voltammetry (CV) curves of pure PBFDO films and PAIh with different IL contents.

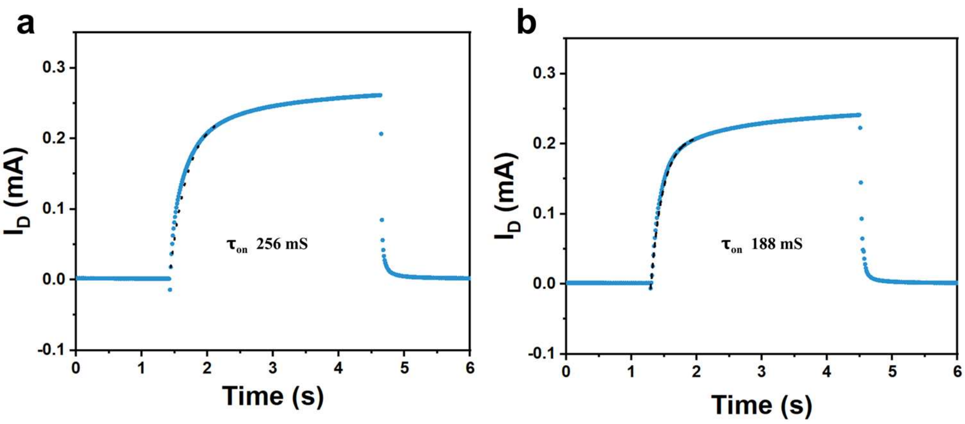

Figure S14 Response time of OECTs fabricated by a) pure PBFDO films and b) PAIh hydrogels.

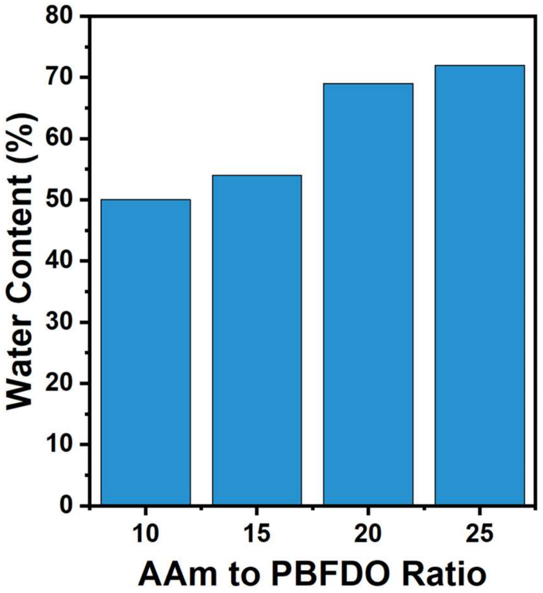

Figure S15 Water content of PAIh with different AAm content.

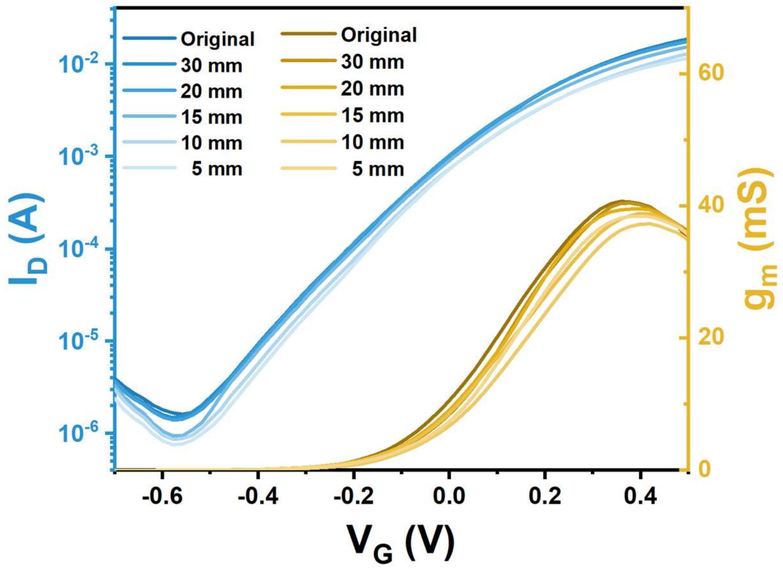

Figure S16 Bending stability of the hydrogel OECT at different bending radius.

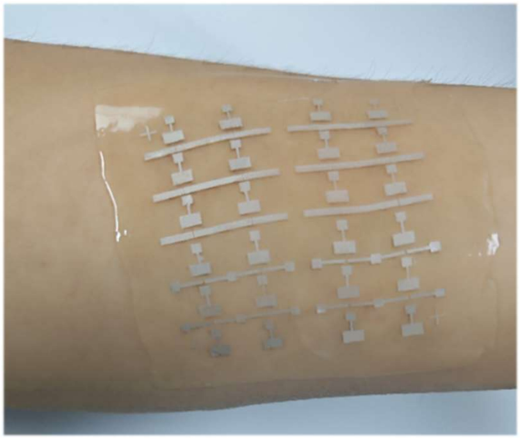

Figure S17 Photograph of the hydrogel OECT array adhered to a person's forearm.

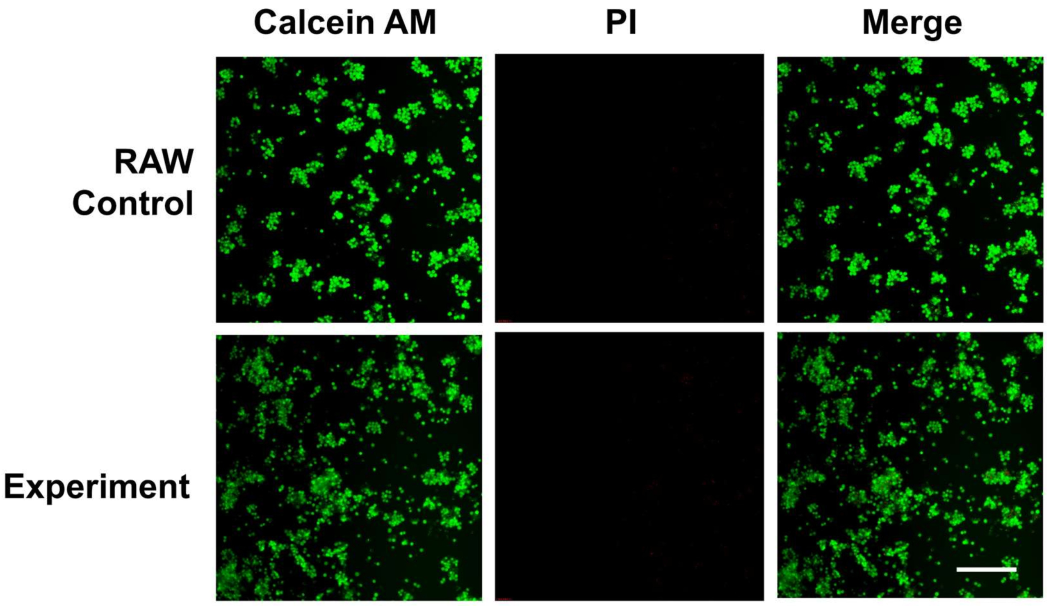

Figure S18 Live/dead stain after 72 hours culture of RAW264.7 cell using hydrogel OECT extracts.

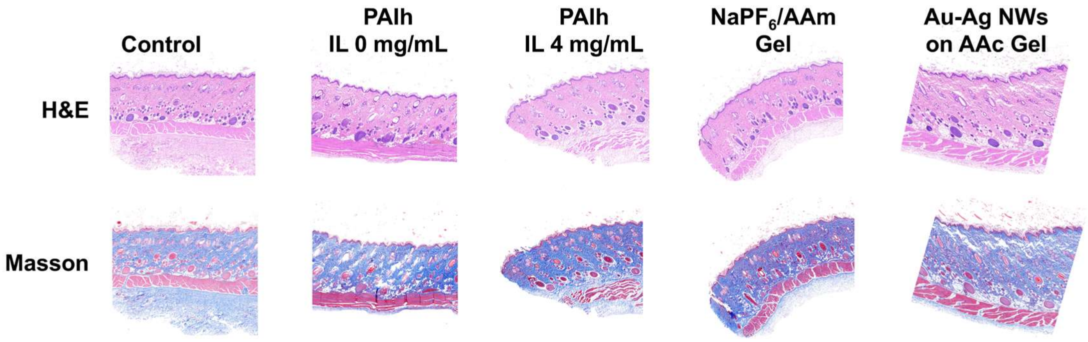

Figure S19 H&E and Masson-stained images of the skin tissues collected 4 weeks after subcutaneous implantation of OECT devices in rats, including all structural components such as PAIh, Ag-Au NWs electrodes, and $NaPF_6$/PAAm hydrogel electrolyte.

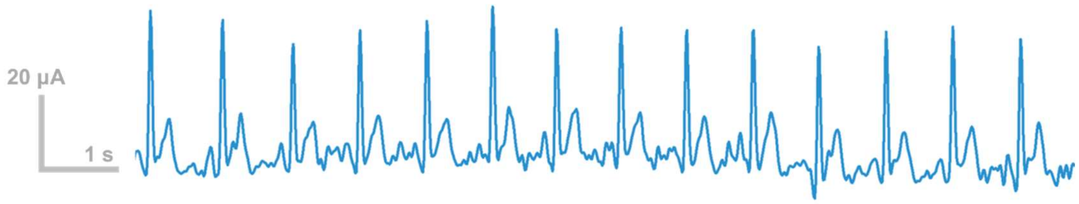

Figure S20 Human ECG signals recorded via hydrogel OECTs.

Table S1 Comparison of OECT performance using Ag-Au NWs and Cr/Au thin film electrodes

Electrode type	Transconductance (mS)	Threshold voltage (V)	I_{ON}/I_{OFF} ($\times 10^3$)
Ag-Au NWs	44.52 ± 3.71	0.382 ± 0.013	1.37 ± 0.35
Cr/Au thin-film	39.96 ± 1.93	0.372 ± 0.012	1.41 ± 0.24

Table S2 Signal-to-noise ratio (SNR) of physiological signal monitoring results

Signal type	Measurement condition	SNR
Human EOG	Adhere to the skin	21.7 dB
Human ECG	Adhere to the skin	16.4 dB
Rat nociceptive signals	Subcutaneous implantation	13.3 dB
Rat ECG	Subcutaneous implantation	7.28 dB
Rat ECG after one week of implantation	Subcutaneous implantation	5.86 dB
